# First confirmed report of *Nassarius sinarum* (Mollusca, Gastropoda) in Korea

**DOI:** 10.3897/BDJ.11.e99661

**Published:** 2023-06-17

**Authors:** Sungha Cho, Inho Yang, Jong Seong Khim, Jinsoon Park

**Affiliations:** 1 Department of Convergence Study on the Ocean Science and Technology, Korea Maritime and Ocean University, Busan, South Korea Department of Convergence Study on the Ocean Science and Technology, Korea Maritime and Ocean University Busan South Korea; 2 Department of Oceanographic Survey, Geosystem Research Corp., Gunpo, South Korea Department of Oceanographic Survey, Geosystem Research Corp. Gunpo South Korea; 3 School of Earth and Environmental Sciences and Research Institute of Oceanography, Seoul National University, Seoul, South Korea School of Earth and Environmental Sciences and Research Institute of Oceanography, Seoul National University Seoul South Korea

**Keywords:** COI, DNA barcoding, estuary, Gastropoda, H3, scanning electron microscope

## Abstract

**Background:**

The marine gastropod mollusc *Nassarius sinarum* has attracted attention due to its status as a potential invasive species and the ecological impact it may have on local environments and the fishing industry. It was observed exclusively within China initially, but its distribution now seems to have expanded into Japan and Korea. Accurate identification of *N. sinarum*, particularly in its juvenile stage, is vital for understanding its ecological influences and distribution patterns.

**New information:**

This study represents the first comprehensive analysis of *N. sinarum* samples from Korea. It includes morphological examination, scanning electron microscopy images and molecular sequencing. Two live specimens were collected from the Yeongsan River estuary in Korea and their morphological features were analysed and compared to those of samples from China and Japan. The samples’ species were confirmed by molecular identification, based on cytochrome c oxidase subunit I (COI) and histone H3 (H3) genetic markers.

It was observed that juvenile *N. sinarum* shells lack key species-characteristic morphological traits, such as a thick outer lip and diminishing axial ribs. However, COI marker-based molecular identification affirmed that these Korean specimens were *N. sinarum*. The H3 region was registered with the National Center for Biotechnology Information (NCBI) for the first time. Phylogenetic analysis of the H3 region did not resolve species distinctions within the *Nassarius*, suggesting that the H3 marker is not suitable for species identification within this genus. In this context, multiple genetic markers, when used appropriately, can also be applied to genus-level searches, enhancing species identification accuracy and reducing misidentification.

The sequences provided in this study can serve as a valuable reference for future DNA barcoding research. Additional samples and surveys should be conducted through collaborative efforts amongst national and institutional organisations to further clarify the ecological status of *N. sinarum* and to investigate its distribution and potential impact around East Asia. Finally, a new Korean name, (No-lan-jul-job-ssal-mu-nui-go-dung; 노란줄좁쌀무늬고둥) has been proposed for *N. sinarum*.

## Introduction

The Nassariidae constitute a globally-occurring family of shelled gastropods with a broad habitat range spanning from the intertidal zone to deep waters (1000 m depth) and with a distribution that encompasses temperate, subtropical, tropical and cold waters (Cernohorsky 1972). Typically, Nassariidae species, commonly referred to as nassa mud snails, inhabit tidal flats and they exhibit carnivorous and carcass-scavenging consumption behaviour (Okutani 2017). With more than 600 species belonging to at least 23 genera, the Nassariidae have a diverse taxonomic representation (MolluscaBase eds. 2022). Despite the wide distribution of nassa mud snails globally, research indicates that only 24 species across five Nassariidae genera have been identified in Korea (NIBR 2019). Therefore, further systematic investigations of Nassariidae species within Korea are needed to gain a comprehensive understanding of the diversity and distribution of this family in the region. 

For more than a century and a half after its initial discovery, the Nassariidae species *Nassarius sinarum* (Philippi 1851) was observed solely in the China Sea (Cernohorsky 1984). Mito and Uesugi (2004) then identified *N. sinarum* as an alien species that was introduced or established in Japan, suggesting that it may be an exotic species introduced from mainland China to Japan. The potential negative impact of *N. sinarum* on ecosystems as a non-native species is currently being evaluated in Japan using the Invasive Alien Species System (IAS). Lee et al. (2010) warned that *N. sinarum *is likely to be introduced to Korea due to its successful introduction and emerging establishment in neighbouring Japan. An observation of *N. sinarum* in Suncheon Bay (Korea) was recorded in June 2009, albeit with its name misspelled as *N.* (*Zeuxis*) *sinarus*; however, it was not added to the national species list of Korea (Hong et al. 2010).

The sole documentation of *N. sinarum* in Korea prior to the present study was a solitary photograph without morphological verification of the specimen, DNA barcoding, a record of whether the specimen was a whole organism or merely a shell or the vouchering of the specimen to a museum. Furthermore, there were morphological discrepancies compared to previously-reported *N. sinarum* individuals, particularly with respect to the aperture. Thus, the purported initial identification of *N. sinarum* in Korea requires more accurate evidence to be collected. These inadequacies may account for the species' absence in Korean records.

In the present study, specimens of *N. sinarum* were collected from the Yeongsan River estuary in Korea. A detailed assessment of *N. sinarum* was conducted, including morphological description and verification, detailed structural and scanning electron microscopy and molecular verification. *Nassarius* species are usually distinguished, based on shell morphology, especially shell sculpture. However, this distinction might be imprecise and lead to confusion (Li et al. 2010), implying that DNA barcoding is an essential tool for accurate species-level identification (Trivedi et al. 2016). In our investigation, we utilised two genetic markers: the mitochondrial gene cytochrome c oxidase subunit I (COI), which is widely conserved in invertebrates, including gastropods; and the nuclear gene histone H3 (H3), which is known for its utility in revealing deep nodes in phylogenetic tree analyses (Latiolais et al. 2006, Ayyagari and Sreerama 2019). This study provides a formal report and description of *N. sinarum* in Korean waters and contributes to the taxonomic knowledge of extant Nassariidae.

## Materials and methods

### Sample preparation

In June 2022, two *Nassarius sinarum* specimens were obtained from the Yeongsan River estuary, situated in the southwest coastal region of the Korean Peninsula. Following collection, the specimens were preserved immediately at -80°C in a 50 ml conical tube containing seawater from the site to minimise any alterations to their genetic material. This preservation method was adhered to scrupulously to prevent any adverse pre-extraction effects on DNA.

The first specimen (labelled as "A") was subjected to analysis of muscle tissue, which was obtained after breaking its shell for DNA examination. Due to the specimen's diminutive size, extensive shell damage was necessary to isolate muscle tissue for genetic analysis. Subsequently, a sequence file for sample A was submitted to the NCBI, a repository for DNA barcode records related to GenBank data. The submission, which included an extensive data file, underwent a meticulous review process employing BankIt tools to ensure proper formatting, sequence quality and the absence of potential contamination. The data file contained details regarding the organism, organelles, isolation source, country of origin, coordinates, collection date, collectors and identifiers. The submission also included a trace file that indicated the primers used, sequence directionality and molecular marker information. COI and H3 sequences were edited, aligned in FASTA format, assigned a Sample ID and uploaded to GenBank. The specimens' shells were inspected under a Leica S9D stereomicroscope, equipped with a K3C camera.

To prepare it for scanning electron microscopy observation, the second specimen (labelled as "B") was treated with a 10% sodium hydroxide (NaOH) solution at 40°C for 48 hours and then rinsed thoroughly with distilled water. The shells then underwent five consecutive ultrasonic cleaning cycles, each lasting 10 minutes, before being dried completely in a desiccator at 40°C and mounted on stubs for gold coating and scanning electron microscopy analysis. The examination was conducted using a MIRA-3 Field Emission Scanning Electron Microscope (Tescan, Brno, Czech Republic) with aluminium stubs secured with carbon tape. Upon completion of scanning electron microscopy imaging, the pristine *N. sinarum* shell of Sample B was deposited at the Korea Marine Biodiversity Research Institute (MABIK, MO00184324). DNA analysis could not be performed on Sample B due to the requirement of shell crushing for DNA extraction and the dissolution of all organic matter for scanning electron microscopy imaging.

### Primer sequences

We used a HiGene Genomic DNA prep kit (Biofact, Daejeon, Korea) to isolate genomic DNA from 2 mg muscle tissue samples. Extracted DNA was subjected to bidirectional sequencing, focusing on two DNA regions for amplification: COI and H3. COI was amplified with forward and reverse primers:

LCO1490 (5’-GTAAAACGACGGCCAGTGGTCAACAAATCATAAAGATATTGG-3’) and

HCO2198 (5'-CAGGAAACAGCTATGACTAAACTTCAGGGTGACCAAAAAATC A-3')

(Folmer et al. 1994). H3 gene amplification was performed with primers:

H3aF (5'-ATGGCTCGTACCAAGCAGAC(ACG)GC-3') and

H3aR (5'-ATATCCTT(AG)GGCAT(AG)AT(AG)GTG AC-3') (Colgan et al. 1998).

### Target PCR mixture

For COI gene amplification, a 25 μl reaction mixture was prepared consisting of 2 μl DNA template, 12.5 μl 2X Lamp Taq PCR Master mix (Biofact) and 2 μl of forward and reverse primers mixed together (each 10 pmol/μl). Similarly, H3 was amplified with a 25 μl reaction mixture containing 2 μl genomic DNA template, 2.5 μl 10X Lamp Taq buffer, 0.5 μl 10 mM dNTP mix, 0.4 μl Lamp Taq PCR Master mix (Biofact) and 2 μl of forward and reverse primers mixed together (each 10 pmol/μl). The final volume of each of these two reaction mixtures was adjusted to 25 μl with distilled water.

### PCR condition and sequencing

The thermocycling protocol involved separate PCR procedures for COI and H3. The COI PCR was comprised of an initial 120-s denaturation step at 95°C, followed by forty 20-s denaturation cycles, a 40-s annealing step at 45°C, a 60-s extension step at 72°C and a final 300-s extension step at 72°C. The H3 PCR consisted of an initial 120-s denaturation step at 95°C, followed by forty 20-s denaturation cycles, a 40-s annealing at 48°C, a 60-s extension step at 72°C and a final 180-s extension step at 72°C. Amplified PCR products were visualised using agarose gel electrophoresis (1% agarose gel) under UV light after staining with EcoDye™ DNA Staining Solution (Biofact). Sequencing reactions were carried out with BigDye™ Terminator V.3.1 Cycle Sequencing Kits (ABI, Waltham, USA) and all PCR products were sequenced using a 3730XL DNA Analyzer (ABI) at the Biofact sequencing facility.

### Phylogenetic analyses

In the construction of the COI phylogeny, we employed the 10 sequences of *N*.* sinarum* available on the NCBI database, along with 29 sequences from *Nassarius* species, culminating in a collective set of 43 sequences, inclusive of the three outgroup species. When it came to the H3 sequence of *N. sinarum*, we were limited by the fact that no other sequences from the same species were available, given our pioneering registration of it with the NCBI. Therefore, our analysis solely incorporated the sequences from the 29 chosen *Nassarius* species, bringing the total to 33 sequences when considering the three outgroup species. We have chosen these 29 *Nassarius* sequences from species that have sequences available for both COI and H3, thereby ensuring a consistent comparison under uniform conditions. Three species served as outgroups: *Volutharpa perryi*, *Buccinum pemphigus* and *Neptunea cumingi* (Yang et al. 2019). Multiple sequence alignments were executed using ClustalW (Thompson et al. 1994) in BioEdit ver. 7 (Hall 1999). Genetic distances were determined and a Maximum Likelihood tree was constructed in MEGA 11 (Tamura et al. 2021) following the Kimura-2-parameter (K2P) model with 1,000 bootstrap replications (Kimura 1980). The number of branches (> 70) corresponds to bootstrap probabilities in 1,000 bootstrap replications. The analysis conditions for COI and H3 were identical to each other.

## Data resources

The data underpinning the analysis reported in this paper are deposited in the Mendeley Data Repository at http://dx.doi.org/10.17632/zrb238r89g.2.

## Taxon treatments

### Nassarius sinarum

(Philippi, 1851)

1EADFA54-EBC7-5454-B684-64BA036D455D

https://www.marinespecies.org/aphia.php?p=taxdetails&id=560319

#### Synonyms and Combinations

*Buccinum sinarum* (Philippi 1851: v. 8, p. 63)

*Nassa* (*Niotha*) *sinarum* (Philippi (1851): v. 8, p. 63); Tsuchiya (2017): p. 910–917; Okutani (2017): p. 1375.

*Nassarius* (*Zeuxis*) *sinarus* (Philippi (1851): v. 8, p. 63); Cernohorsky (1984): v. 14, p. 289, pl. 31, figs. 10–11; pl. 32, fig. 1; Tamaki et al. (2002): v. 8, no. 2, p. 63–81; Lutaenko et al. (2013): p. 75.

*Nassarius* (*Tritonella*) *semiplicatus* (A. Adams (1852): no. 19, p. 107); Liu (2008): p. 1267.

*Nassarius* (*Zeuxis*) *semiplicatus* (A. Adams (1852): no. 19, p. 107); Zhang and Yang (2010): v. 41, no. 5, p. 791–5.

*Nassarius semiplicatus* (A. Adams (1852): no. 19, p. 107); Zhang and Yang (2010): v. 41, no. 5, p. 791–5.

*Zeuxis semiplicata* (A. Adams (1852): no. 19, p. 107); A. Adams (1852): v. 24, p. 233, pl. 23, figs. 164; Cernohorsky (1984): v. 14, p. 154.

#### Material examined

**Figure 1.**
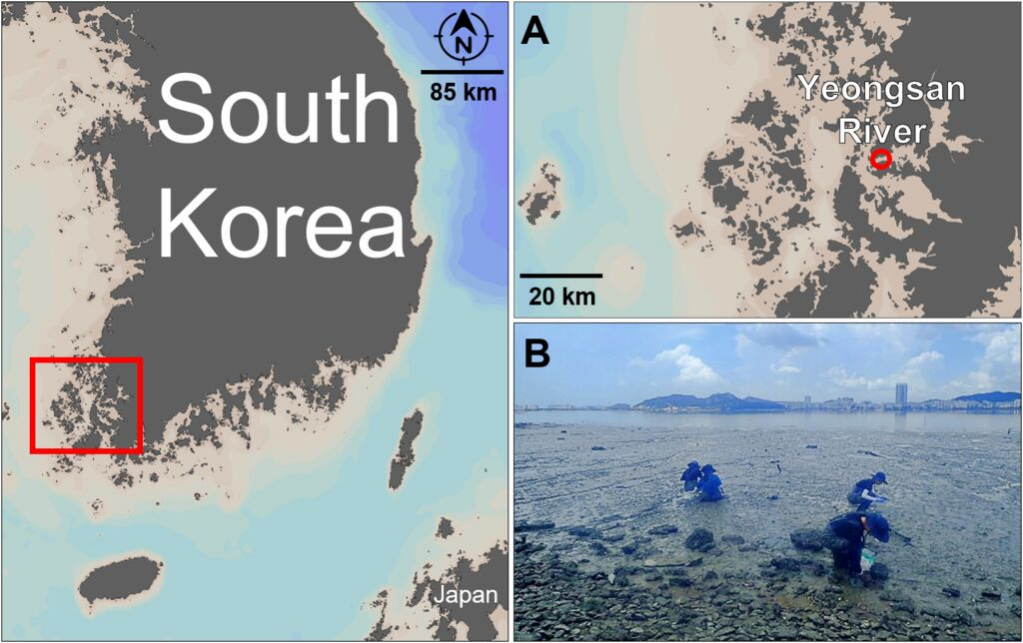
Sample collection site details. **A **The fine-scale location of the collection site was obtained using Ocean Data View (Schlitzer R, 2015, http://odv.awi.de); **B** Photograph of the sampling location at the Yeongsan River, taken in June 2022 and provided by Sungha Cho.

On 16 June 2022, two live specimens were collected from the Yeongsan River estuary at Jeollanam-do, Korea (34°46'48"N, 126°26'31"E), located at 219 Daeburyeok-ro Samho-eup, Yeongam-gun (Fig. 1). The environmental conditions at the time of collection included a water temperature of 24.5°C, dissolved oxygen (DO) levels of 5.69 mg/l, salinity of 33.24 practical salinity units (PSU) and a pH of 8.37. The collector responsible for this sampling was Sungha Cho.

#### Description

**Figure 2.**
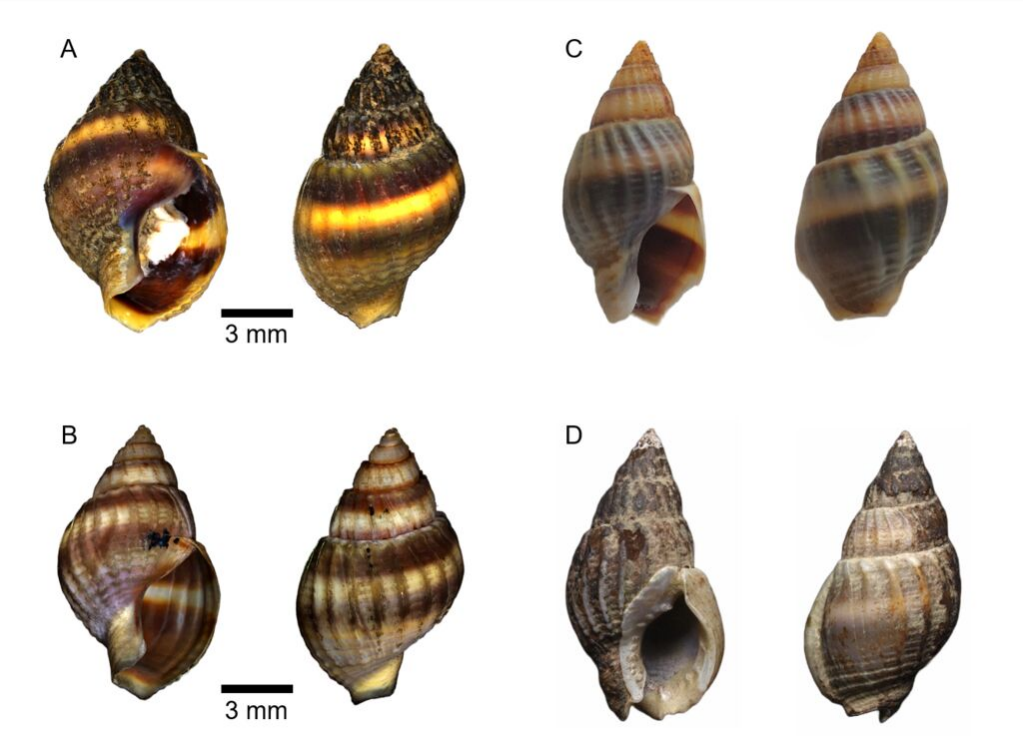
Morphological images of *Nassarius sinarum* from Korea, China and Japan. **A & B** Specimens collected alive in the estuary at the mouth of the Yeongsan River in Korea (this study) and considered to be juvenile shells; apertural and dorsal views were photographed; **C **Specimen collected alive in China; **D** Specimen collected alive in the mudflats of Japan; morphological features of the apertural and dorsal views suggest it is an adult shell. Scale bars for A and B are 5 mm; scale bars for C and D are not provided. For original images and data, consult Suppl. material 1.

**Figure 3.**
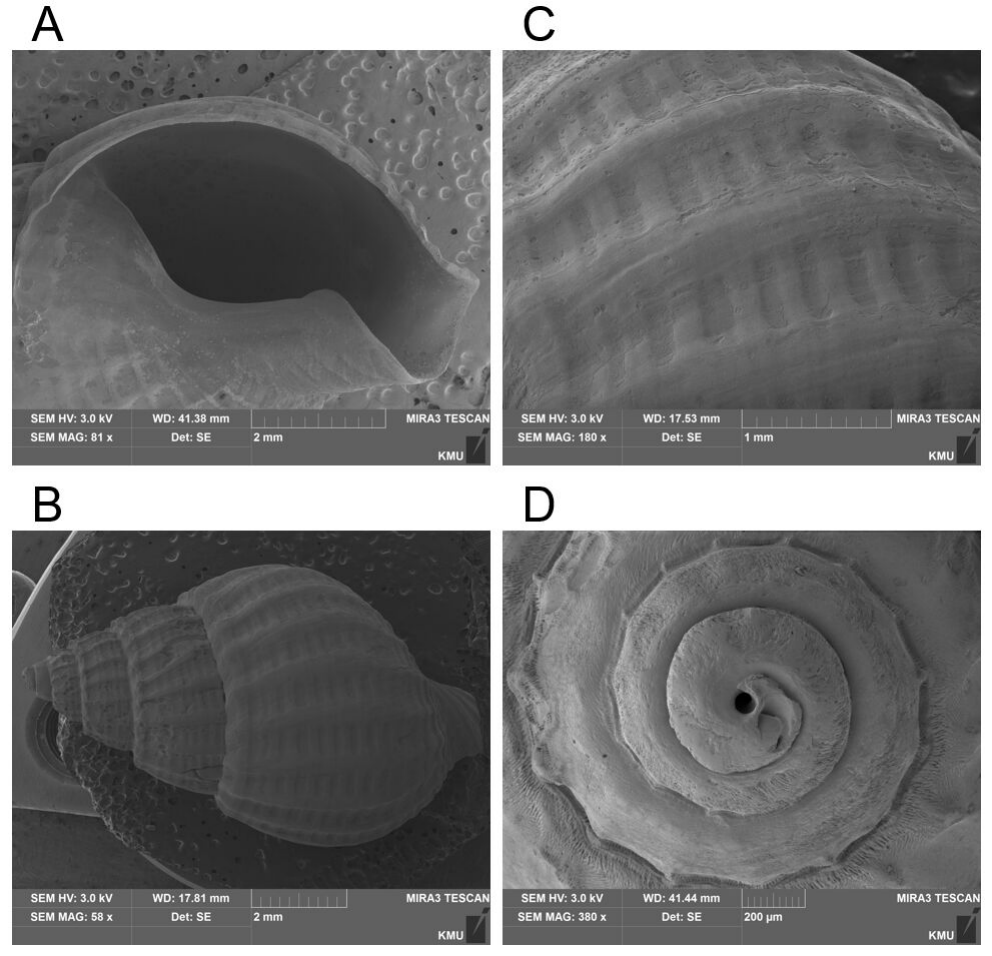
Scanning electron microscope images of the shell of *Nassarius sinarum *Specimen B, collected in this study. **A **Morphology, characterised by the absence of a developed callus and tooth in the aperture; **B **Axial ribs exhibiting a consistent appearance as they extend to the outer lip; **C** Body whorl sculpture; **D** Protoconch. Scale bar A & B = 2 mm, C = 1 mm, D = 200 μm. For additional scanning electron microscopy images, please refer to Suppl. material 1.

Sample A, exhibiting a shell length of 14 mm and Sample B, with a shell length of 15 mm, are not fully developed individuals in comparison to the known maximum size of *N*. *sinarum*, which reaches up to 20 mm (Fig. 2). The undeveloped outer lip at the base further indicates that these specimens might be juveniles or subadults (Fig. 3). Both individuals A and B seem to be at the same developmental stage. Samples A and B, collected in this study, were photographed in both apertural and dorsal views to facilitate morphological comparisons. Sample C, collected in China, is depicted in apertural and dorsal views and is considered a juvenile shell, similar in growth stage to Samples A and B from Korea, due to the absence of a developed thick outer lip (Lynio 2023). Nonetheless, the axial ribs become progressively thinner as they approach the outer lip, suggesting that the specimen may be at a more mature developmental stage. Specimen D, collected in Japan, appears to be an adult shell, based on morphological features observed in apertural and dorsal views and exhibits a greyish-brown hue with pale spiral bands (Magokorogai 2013). The axial ribs are obtuse, spaced and knobbly at the shoulders, but become obsolete towards the base. The aperture is white inside and the callus on the columellar lip is demarcated, bearing one tooth at the posterior end. The anal canal is narrow and the inner wall of the outer lip is denticulated. In contrast, Korean Samples A and B, believed to be juveniles, display an aperture with uniform colouration and lack callus and tooth formation throughout the shell. Specifically, in the adult body whorl, the axial ribs weaken towards the outer lip, ultimately becoming nearly smooth, which is a key distinguishing feature of *N. sinarum*, yet not observed in juvenile shells. Limited photographs of *N. sinarum* are available for comparison and those that exist are fragmentary.

#### Distribution

Initially, *N. sinarum* was thought to be exclusive to the Yangtze River in China (Cernohorsky 1984). However, in 2004, its presence was later documented in Japan, where it was classified as an alien species (Mito and Uesugi 2004). Although Hong et al. (2010) reported observing it in Suncheon Bay, Korea in June 2009, the species' introduction remained uncertain due to a lack of corroborating data. This study documents the collection of live *N. sinarum* specimens from the Yeongsan River estuary in Korea, confirming that the species now inhabits Korea, China and Japan.

#### Molecular data

**Figure 4.**
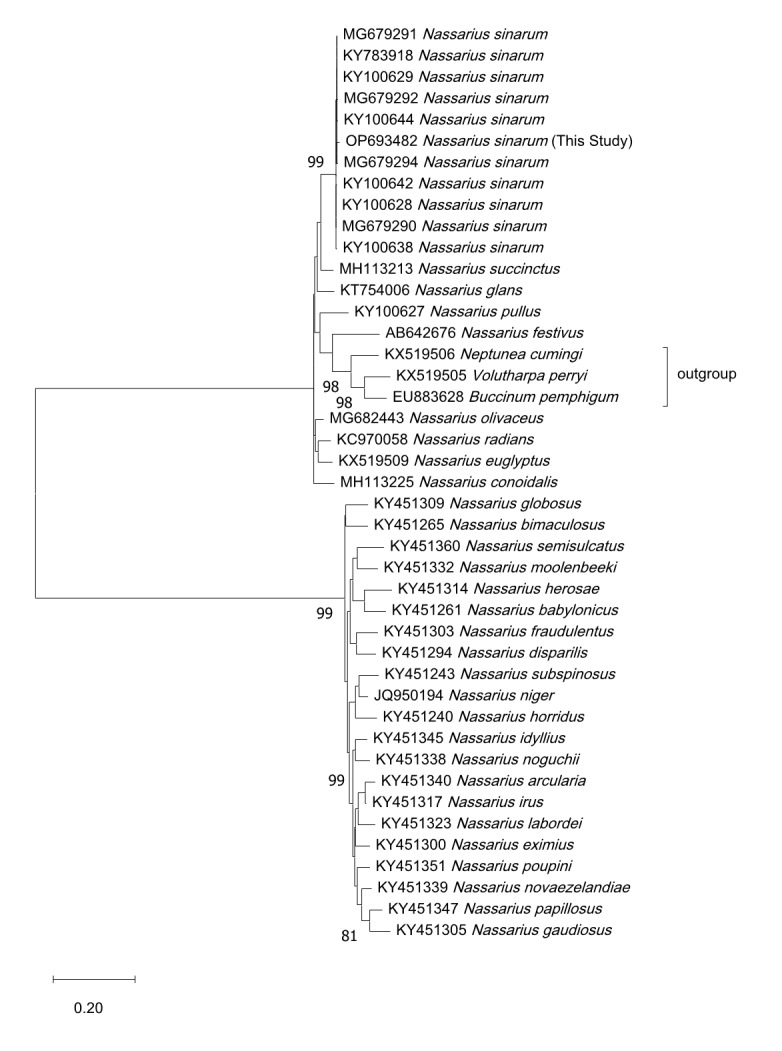
Maximum Likelihood tree of COI sequences of *Nassarius sinarum* collected in Korea. Bars represent genetic distances, letters preceding scientific names are NCBI accession numbers. A phylogenetic tree analysis conducted with the COI region demonstrates that *N. sinarum* has a distinctive species-indicative COI sequence.

**Figure 5.**
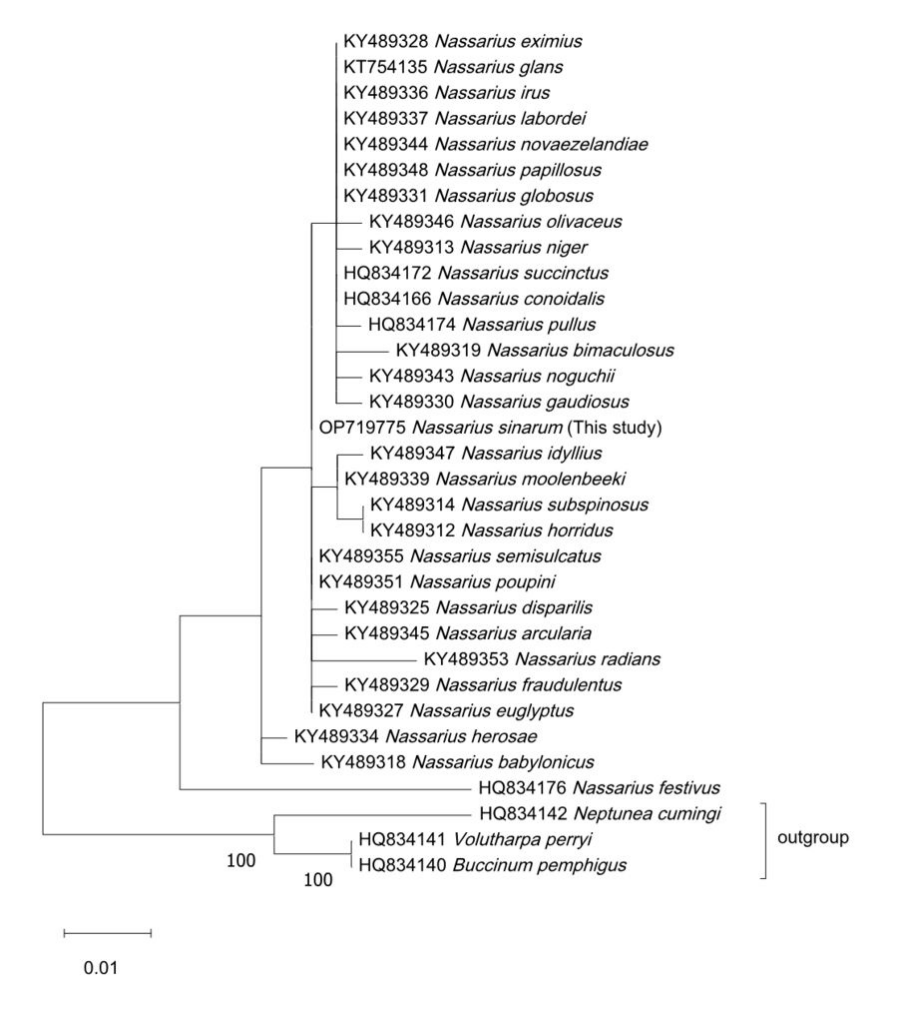
Maximum Likelihood tree of H3 sequences of *Nassarius sinarum* collected in Korea. Bars represent genetic distances, letters preceding scientific names are NCBI accession numbers. A phylogenetic tree analysis conducted with the H3 region reveals that H3 sequences of *N. sinarum* cannot be used to clearly distinguish it from other *Nassarius *species.

The sequence files were submitted to the NCBI and assigned accession numbers: OP693482 (660 bp) for COI and OP719775 (372 bp) for H3. The NCBI Basic Local Alignment Search Tool (BLAST) analysis demonstrated that our *N. sinarum* COI sequence matches that previously registered for *N. sinarum*, exhibiting 98.48–99.69% sequence similarity (Table 1). *N. succinctus* was the nearest species, with 93.86% percent similarity. Our sequence's similarity as *N. sinarum* was further confirmed by a Maximum Likelihood tree, which was analysed for percentage similarity with the top 10 sequences retrieved from NCBI BLAST. Outgroups (*Volutharpa perryi, Buccinum pemphigus *and *Neptunea cumingi*) were included to improve clustering and branch support (Yang et al. 2019). To bolster the robustness of the phylogenetic analysis, we incorporated an additional 29 species from the *Nassarius*. The close clustering of *N. sinarum* within the phylogenetic COI tree suggests species consistency; no sequence differences were observed within species or between countries (Fig. 4). An H3 phylogenetic analysis was performed for more accurate identification, using the same conditions as were used for the COI analysis. As the H3 region of *N. sinarum* is novel to the NCBI database, species-level comparisons were not feasible. The H3 phylogenetic tree encompassed difficulties in distinguishing amongst species within the same genus (Fig. 5). Thus, it appears that H3 is not a suitable marker for differentiating species within the *Nassarius*.

#### Ecology and habitat

*Nassarius*, commonly known as nassa mud snails (USA) or dog whelks (UK), is a genus of small to medium-sized marine gastropod molluscs in the Nassariidae characterised by their scavenging behaviour. These shelled gastropods inhabit mud flats and sand flats, have a global distribution and have a diverse habitat range that spans from the intertidal zone to deep waters (depths of at least 1000 m) in temperate, subtropical, tropical and cold-water environments. *Nassarius* species are highly active scavengers, feeding on crabs and carrion, such as dead fish. They often burrow into marine substrates, waiting with only their siphon exposed until detecting nearby food sources. Consistent with these ecological traits, *N. sinarum* inhabits both sandy and muddy mudflats and occupies various environments within the intertidal zone (Okutani 2017).

#### Remarks

Iwasaki et al. (2004) documented damage related to the presence of *N. sinarum*, including predation on fish (e.g. gobies) captured in fishing nets, in Japan's Saga and Kumamoto Prefectures. The species is believed to have been introduced to Japan through the importation of edible shellfish from China. Currently classified as an alien species in Japan, *N. sinarum*'s status as a harmful invasive species is still under evaluation by Japan's IAS (Mito and Uesugi 2004). Nevertheless, purported negative impacts on the fishing industry suggest that this species warrants attention and monitoring.

## Discussion

This study is the first to present a morphological analysis, inclusive of scanning electron microscopy images and molecular sequencing, of *Nassarius sinarum* individuals collected in Korea. The main identification features of *N. sinarum* include a thick outer lip and axial ribs that diminish towards the outer lip. Typically, juvenile *Nassarius* shells lack a fully developed callus, which leads to confusion in species identification (Barash and Danin 1977). The specimens collected in this study appeared to be juvenile shells lacking species-characteristic morphological traits. However, molecular identification confirmed their classification as *N. sinarum*, allowing us to provide a characterisation of the species at a juvenile stage (Fig. 2).

The COI region of DNA, a universal barcode for gastropods, can be used to identify *N. sinarum* specimens. We registered the H3 region of the *N. sinarum* genome with NCBI for the first time and conducted a genus-level phylogenetic analysis. The phylogeny of the COI region formed a clear clade of COI-sequence conserved species with no regional variation amongst specimens in Korea, China and Japan (Fig. 4). However, our phylogenetic analysis of the H3 region across species of the Nassariidae revealed lack of distinction across *Nassarius* species, indicating that the H3 region is not useful for identifying Nassariidae species (Fig. 5). Further detailed ecological studies are required to investigate these molecular biological findings more comprehensively. In gastropod DNA regions, similar numbers of sequences have been registered for H3 (6,600), 18S (7,300), ITS1 (7,200), 5.8S (14,700), ITS2 (6,100) and 28S (18,900); these sequences can be used to supplement the COI (103,900) region. We continue to recommend employing DNA analyses with multiple genetic markers as a safeguard, given that the use of multiple markers enhances species identification accuracy and reduces misidentification. The functionality of H3 in *Nassarius* species is uncertain. The sequences provided in this study, sourced from samples deposited in the NCBI GenBank, constitute valuable references for future DNA barcoding research.

Cernohorsky (1984) had considered *N. sinarum*’s habitat range to be restricted to China. Some 30 years later, Mito and Uesugi (2004) reported it as an alien species in Japan that had been introduced from China. Lee et al. (2010) predicted that *N. sinarum* would be introduced into Korea and Hong et al. (2010) recorded purported observations, but the present morphological analysis indicates that Hong et al. had reported without sufficient evidence (Suppl. material 1). Currently, the Japanese IAS is assessing whether *N. sinarum* is an invasive species in Japan and direct damage to fisheries has been reported (Iwasaki et al. 2004, Mito and Uesugi 2004). To clarify the ecological status of *N. sinarum*, we suggest that surveys for more samples from more sites be conducted through national and institutional cooperation. In this study, N. sinarum was collected from the Yeongsan River estuary, indicating that this species may be spreading into numerous estuarine ecosystems and brackish water lakes in Korea. For the first time in Korea, we report the presence of *N. sinarum*, which was confirmed by morphological and molecular analytical data and we propose the new Korean name “No-lan-jul-job-ssal-mu-nui-go-dung” (노란줄좁쌀무늬고둥) for this species.

## Supplementary Material

XML Treatment for Nassarius sinarum

18F12982-A1F0-5A20-9DDC-872C77273B1E 10.3897/BDJ.11.e99661.suppl1 8054490 Supplementary material 1 High-Resolution Images and Scanning Electron Microscopy (SEM) of *Nassarius sinarum *Shells Data type High-resolution original size images, SEM images Brief description Original high-resolution images of live shells, dead shells and YSI; SEM images depicting sculpture details, protoconch, spire cross-section, columellar folds and posterior canal of *Nassarius sinarum* shells. File: oo_856865.url https://binary.pensoft.net/file/856865 Sungha Cho, Inho Yang, Jong Seong Khim, Jinsoon Park

## Figures and Tables

**Table 1.** The top 10 results for each of the NCBI BLAST searches, conducted on the COI and H3 regions of *Nassarius sinarum,* are presented in descending order of percentage similarity.

**COI**			**H3**		
**BLAST search**	**Accession No. (bp)**	**Percent Similarity**	**BLAST search**	**Accession No. (bp)**	**Percentage Similarity**
*Nassarius sinarum*	KY100629 (642)	99.69%	*Nassarius siquijorensis*	HQ834169 (354)	100.00%
*Nassarius sinarum*	MG679291 (601)	99.67%	*Nassarius *cf. *comptus*	KY489320 (331)	100.00%
*Nassarius sinarum*	KY100644 (642)	99.53%	*Nassarius velatus*	LC384075 (291)	100.00%
*Nassarius sinarum*	KY100628 (642)	99.53%	*Nassarius velatus*	LC384073 (291)	100.00%
*Nassarius sinarum*	MG679294 (601)	99.50%	*Nassarius velatus*	LC384072 (291)	100.00%
*Nassarius sinarum*	MG679292 (601)	99.50%	*Nassarius velatus*	LC384074 (283)	100.00%
*Nassarius sinarum*	MG679290 (601)	99.50%	*Nassarius conoidalis*	HQ834165 (354)	99.72%
*Nassarius sinarum*	KY783918 (709)	99.39%	*Nassarius *sp. 279	KY489360 (331)	99.70%
*Nassarius sinarum*	KY100642 (642)	99.38%	*Nassarius arcularia*	KY489345 (331)	99.70%
*Nassarius sinarum*	KY100638 (642)	99.38%	*Nassarius moolenbeeki*	KY489339 (331)	99.70%
